# The CBIT+TMS Trial: study protocol for a two-phase randomized controlled trial testing neuromodulation to augment behavior therapy for youth with chronic tics

**DOI:** 10.21203/rs.3.rs-2949388/v1

**Published:** 2023-06-13

**Authors:** Christine Conelea, Deanna Greene, Jennifer Alexander, Kerry Houlihan, Sarah Hodapp, Brianna Wellen, Sunday Francis, Bryon Mueller, Timothy Hendrickson, Angela Tseng, Mo Chen, Mark Fiecas, Kelvin Lim, Alexander Opitz, Suma Jacob

**Affiliations:** Department of Psychiatry and Behavioral Sciences, Masonic Institute for the Developing Brain, University of Minnesota, USA; Department of Cognitive Science, University of California San Diego, USA; Department of Psychiatry and Behavioral Sciences, Masonic Institute for the Developing Brain, University of Minnesota, USA; Department of Psychiatry and Behavioral Sciences, Masonic Institute for the Developing Brain, University of Minnesota, USA; Department of Psychiatry and Behavioral Sciences, Masonic Institute for the Developing Brain, University of Minnesota, USA; Department of Psychiatry and Behavioral Sciences, Masonic Institute for the Developing Brain, University of Minnesota, USA; Department of Psychiatry and Behavioral Sciences, University of Minnesota, USA; Department of Psychiatry and Behavioral Sciences, University of Minnesota, USA; University of Minnesota Informatics Institute, Masonic Institute for the Developing Brain, USA; Department of Psychiatry and Behavioral Sciences, Masonic Institute for the Developing Brain, University of Minnesota, USA; Non-invasive Neuromodulation Lab, Brain Conditions, MnDRIVE Initiative, University of Minnesota, USA; Department of Psychiatry and Behavioral Sciences, University of Minnesota, USA; Neuroscience Program, Research Department, Gillette Children’s Specialty Healthcare, USA; School of Public Health, Division of Biostatistics, University of Minnesota, USA; Department of Psychiatry and Behavioral Sciences, University of Minnesota, USA; Department of Biomedical Engineering, University of Minnesota, USA; Department of Psychiatry and Behavioral Sciences, University of Minnesota, USA

**Keywords:** Tourette, tic, transcranial magnetic stimulation, behavior therapy, pediatric, neuromodulation

## Abstract

**Background:**

Comprehensive Behavioral Intervention for Tics (CBIT) is a first-line treatment for tic disorders that aims to improve controllability over tics that an individual finds distressing or impairing. However, it is only effective for approximately half of patients. Supplementary motor area (SMA)-directed neurocircuitry plays a strong role in motor inhibition, and activity in this region is thought to contribute to tic expression. Targeted modulation of SMA using transcranial magnetic stimulation (TMS) may increase CBIT efficacy by improving patient ability to implement tic controllability behaviors.

**Methods:**

The CBIT+TMS trial is a two-phase, milestone driven early-stage randomized controlled trial. The trial will test whether augmenting CBIT with inhibitory, noninvasive stimulation of SMA with TMS modifies activity in SMA-mediated circuits and enhances tic controllability in youth ages 12–21 years with chronic tics. Phase 1 will directly compare two rTMS augmentation strategies (1Hz rTMS vs. cTBS) vs. sham in N = 60 participants. Quantifiable, a priori “Go/No Go Criteria” guide the decision to proceed to Phase 2 and selection of the optimal TMS regimen. Phase 2 will compare the optimal regimen vs. sham and test the link between neural target engagement and clinical outcomes in a new sample of N = 60 participants.

**Discussion:**

This clinical trial is one of few to date testing TMS augmentation of therapy in a pediatric sample. Results will provide insight into whether TMS is a potentially viable strategy for enhancing CBIT efficacy and reveal potential neural and behavioral mechanisms of change.

**Trial registration:**

ClinicalTrials.gov Identifier: NCT04578912.

## Administrative information

Note: the numbers in curly brackets in this protocol refer to SPIRIT checklist item numbers. The order of the items has been modified to group similar items (see http://www.equator-network.org/reporting-guidelines/spirit-2013-statement-defining-standard-protocol-items-for-clinical-trials/).

## Introduction

### Background and rationale {6a}

Chronic tics are the primary symptom of Tourette Syndrome and Persistent Motor/Vocal Tic Disorder (1). Chronic tics affect 1–3% of youth (2) and are associated with adverse impacts on functioning, physical pain, diminished quality of life, peer victimization, and a fourfold increased risk of suicide compared to the general population (3–5).

Comprehensive Behavioral Intervention for Tics (CBIT; (6)) is the current gold-standard, first-line treatment for tics (7). Large randomized controlled trials established the superiority of CBIT over supportive therapy in children (8) and adults (9), and meta-analyses show comparable effect sizes for CBIT and antipsychotic medications (10). However, only 52% of children (8) and 38% of adults (9) showed clinically meaningful tic improvement in the original CBIT trials, demonstrating a need for targeted augmentation of CBIT to improve response.

The overarching goal of CBIT is to improve tic controllability –a patient’s ability to voluntarily inhibit tics they find impairing or distressing. During the core CBIT procedure, known as competing response training, patients learn to engage in a competing motor action upon noticing tics or tic antecedents. Tic controllability has been shown to drive CBIT improvement (11) and predict lower tic burden over the course of illness (12). However, many youth lack the foundational tic inhibition ability that CBIT aspires to enhance. For example, quantitative assessment of tic controllability using the Tic Suppression Task (TST; (13)) has shown that only 20% of youth can temporarily fully inhibit tics, while another 20% show no tic change or even tic worsening when attempting suppression (14). Targeted enhancement of tic controllability is therefore one plausible way to boost CBIT response.

Tics are associated with dysfunctional activity in cortico-striatal-thalamo-cortical circuits (CSTC; (15, 16), including excessive activity in sensorimotor pathways. The supplementary motor area (SMA) is a key CSTC cortical node that plays a strong role in motor inhibition (17). Evidence implicates SMA activity and hyperconnectivity in tics. For example, neuroimaging studies show increased functional connectivity between SMA and successive nodes of the CSTC sensorimotor circuit,(15) including primary motor cortex (M1; (18)(19)). SMA activity is elevated prior to tic execution(15, 18, 20) and during periods of higher tic frequency (18, 21). Further, SMA activity and connectivity have been shown to be significantly correlated with tic severity and complexity (22) and tic premonitory urge severity (23). Finally, SMA shows strong resting state functional connectivity (RSFC) with striatal deep brain stimulation (DBS) sites that are most effective for treating tics (24).

SMA’s extensive connectivity with regions implicated in motor control and its role in tic pathology have made it a leading brain target candidate for repetitive transcranial magnetic stimulation (rTMS; (25)). During rTMS, a pulsed magnetic field is produced by a small coil positioned over a targeted area on the scalp, inducing an electric current in the brain that modulates cortical activity. rTMS paradigms use trains of pulses to induce cortical effects that outlast the duration of stimulation (26). rTMS has been explored as a tic treatment in small trials and case reports (27–30), some of which included children.(31–33) Early rTMS trials targeting premotor and motor cortex showed no effect (29, 30). In contrast, inhibitory stimulation of SMA using 1Hz rTMS has been associated with reduced tic severity in case reports (27) and open label trials (31, 33, 34). However, small randomized trials targeting SMA inhibition with 1Hz rTMS (35), deep TMS with the HBDL coil (28), and continuous theta burst stimulation (cTBS) did not find group-level clinically meaningful change. One 1Hz rTMS study with null effects at 3 weeks of treatment found benefit after an extended dose (total of 6 weeks daily; (35)). In the only study measuring neural correlates of treatment, Wu et al. (32) detected significant decreases in SMA activation and connectivity to bilateral M1 after a 2 day course of cTBS.

Taken together, the literature suggests that rTMS can engage SMA but may be insufficient as a monotherapy for tics. This notion is convergent with a large body of literature demonstrating that neurostimulation effects highly depend on the state of the targeted circuitry (36, 37), leading for calls to improve TMS outcomes with “functional targeting” that combines TMS with behavioral/cognitive engagement of the same circuit being modulated (38). Few trials to date, however, have systematically tested the combination of TMS and psychotherapy.

Given that CBIT engages and relies on SMA-directed circuitry that is atypical in tic disorders, augmenting CBIT with TMS over SMA may potentiate neuroplasticity and increase CBIT efficacy. Accordingly, we designed the CBIT + TMS trial, an NIH/NIMH-funded two-phase, milestone driven early-stage randomized controlled trial. Here we describe the trial protocol.

### Objectives {7}

The overall objective of the CBIT + TMS trial is to test whether augmenting CBIT with inhibitory, noninvasive stimulation of SMA modifies activity in SMA-mediated circuits and enhances tic controllability in young people with tic disorder. The primary objective of Phase 1 is to directly compare two rTMS regimens previously explored as monotherapies in people with tics as a dose-finding strategy (1Hz rTMS and cTBS). Analyses will focus on testing change in neural (fMRI-measured SMA task activation and RSFC) and behavioral targets (tic controllability).

We will proceed to Phase 2 if we meet quantifiable study “Go/No Go Criteria.” A positive “Go” decision to move to Phase 2 will require demonstration of neural change (within-subject ANOVA effect size ≥ η2 = 0.18), safety (≤ 20% rate of adverse events judged to be treatment related), and feasibility (80% of participants are able to complete 80% of treatment sessions). If these criteria are met, we will identify the “optimal” rTMS regimen for further testing in Phase 2 using the following decision rules: 1) if one regimen is superior (p < .05) on one or both neural targets, that regimen wins; 2) if regimens are equivalent for both targets, or if regimens differ by neural target, we prioritize the regimen with significantly better safety and feasibility outcomes (two-tailed t-test of p < .05). If there is still no difference, we will select cTBS as the “winner” since it is a faster, lower-resource demanding approach.

The primary objective of Phase 2 is to replicate and validate the effects of the optimal rTMS regimen and test the link between neural target engagement and functional outcomes (i.e., whether changes in RSFC of SMA circuitry mediate improved tic controllability and decreased tic severity). We will also test the trajectory of tic controllability change across the course of treatment and explore the durability of neural change through 1-month follow-up and of all measured outcomes through 6-month follow-up.

### Trial design {8}

**Figure 1:**
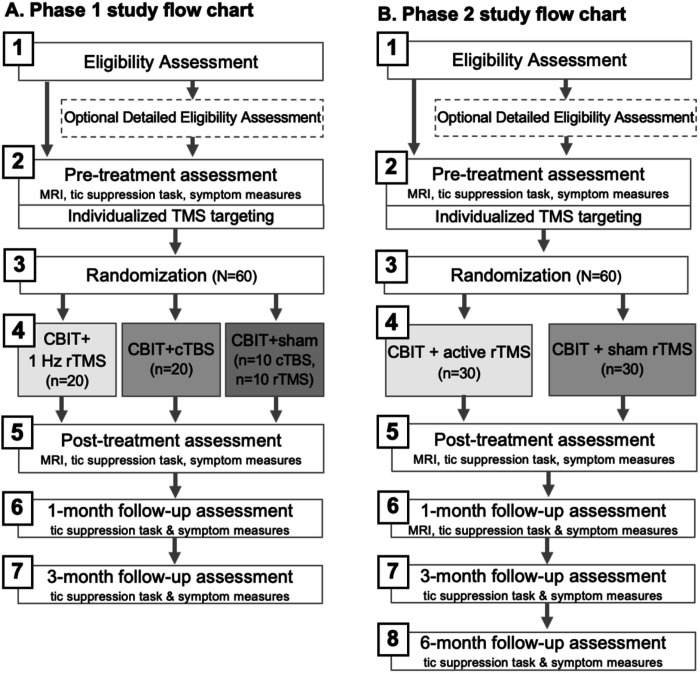
Legend not included with this version

Both Phases are randomized, sham controlled, double masked, parallel group, superiority trials. Phase 1 is a three-arm trial (sham, 1Hz rTMS, or cTBS; n = 20 per group) and Phase 2 is a two-arm trial (sham or active stimulation; n = 30 per group). Randomization in both phases will be blocked on baseline Tic Suppression Task (TST) performance (tic controllability below or above 50%) and medication status (on vs. off). The flow chart of each study phase is shown in Fig. 1.

## Methods: Participants, interventions and outcomes

### Study setting {9}

The study will be conducted in an outpatient clinical-research setting at the University of Minnesota Masonic Institute for the Developing Brain in the United States of America. This center specializes in research and clinical care related to neurodevelopmental conditions. Neuroimaging data will be collected at the University of Minnesota Center for Magnetic Resonance Research.

### Eligibility criteria {10}

#### Participant Eligibility determination.

Interested participants will be initially screened for inclusion/exclusion criteria during a phone call. Medical records relevant to eligibility determination will be obtained from participants directly or, with a signed release of information permission form, from their healthcare provider(s). All eligibility criteria will be confirmed during the pre-treatment assessment and prior to the pretreatment MRI and randomization.

#### Optional detailed eligibility assessment

In some cases, it is possible that the phone screening outcome is unclear but could be clarified with an abbreviated version of the pre-treatment assessment visit focused on the specific eligibility question (e.g., whether tics meet minimum severity threshold). In these cases, a video call visit of up to 1 hour will be scheduled to administer selected measures from the pre-treatment assessment list. For participants deemed eligible, data will be carried-forward when possible to reduce participant burden.

Inclusion Criteria: 1) Age 12–21 years at the time of study enrollment. 2) Current chronic motor and/or vocal tic disorder meeting DSM-5 criteria (1): tics present for ≥ 1 year without a tic-free period of more than 3 consecutive months and tics not due to another medical condition or the direct physiological effects of a substance. 3) At least moderate tic severity, defined as a Yale Global Tic Severity Scale (YGTSS) total score ≥ 14 (≥ 9 for those with motor or vocal tics only), paralleling the criterion used in the CBIT efficacy trials.(39) 4) IQ greater than 70 and 5) Participants and parent/guardian (for minors) with enough English comprehension to provide consent and comprehend study measures and instructions. 6) If taking psychotropic medication, medication status has been stable for 6 weeks with no anticipated changes during the 3 week intervention protocol.

Exclusion Criteria: 1) Medical conditions contraindicated or associated with altered TMS risk profile, including history of intracranial pathology, epilepsy or seizure disorders, traumatic brain injury, brain tumor, stroke, implanted medical devices or metallic objects in the head, current pregnancy or girls of childbearing age not using effective contraception, or any other serious medical condition. 2) Inability to undergo MRI. 3) Left handedness. 4) Active suicidality. 5) Previous diagnosis of psychosis or cognitive disability. 6) Substance abuse or dependence within the past year. 7) Concurrent psychotherapy focused on tics. 8) Currently taking a neuroleptic/antipsychotic medication, as these medications are associated with altered seizure risk.

Individuals who will perform interventions. TMS operators will be staff trained according to recommended guidelines for TMS Technician training (40). CBIT therapists will be individuals with experience delivering behavioral and cognitive-behavioral interventions with youth who have neurodevelopmental disorders. CBIT therapists will undergo training in the study protocol and receive ongoing supervision from a licensed clinician with CBIT expertise.

### Who will take informed consent? {26a}

Consistent with IRB and HIPAA guidelines, trained research staff will obtain informed consent from parents or adult participants (i.e., those age 18 years or older) and assent from minors prior to data collection at the first point of formal assessment (either the optional detailed eligibility assessment or the pre-treatment assessment visit). If any minors turn 18 years old during the course of their participation, they will be re-consented as an adult. Informed consent and assent will be documented electronically using a REDCap platform that is compliant with the US Food and Drug Administration’s (FDA) 21 CFR Part 11 regulations.

### Additional consent provisions for collection and use of participant data and biological specimens {26b}

Participants can optionally consent to allow us to 1) retain tic observation videos for future research and 2) submit deidentified data from this study to the National Institute of Mental Health Database (NDA) at the NIH.

## Interventions

### Explanation for the choice of comparators {6b}

cTBS and conventional 1Hz rTMS are thought to have comparable effects on cortical excitability (41, 42) and similar safety profiles in pediatric samples (43, 44), though they have not previously been compared head-to-head in a pediatric clinical trial. TBS has particular advantages for a pediatric population including much shorter stimulation duration (i.e., 2–3 min for TBS vs. 20–30 min for rTMS) and lower stimulation intensity (44). Sham stimulation enables a placebo comparator to the active conditions.

### Intervention description {11a}

Participants will receive 10 treatment sessions, delivered daily on weekdays for two weeks. Treatment sessions will consist of TMS first, directly followed by CBIT sessions.

#### TMS protocol.

Stimulation will be delivered using a Magstim Super Rapid2 stimulator (Magstim Company Ltd, UK). A Magstim air-cooled 70mm figure-eight coil will be used for motor threshold determination and active TMS conditions. Sham stimulation will use the Magstim sham air-cooled coil, which produces auditory signals identical to an active coil but contains a mu-metal shield that diverts the majority of the magnetic flux such that a minimal (< 3%) magnetic field is delivered to the cortex.

Resting Motor Threshold (RMT) will be determined prior to the first TMS intervention session and used to calculate stimulation intensity for all TMS sessions. RMT is defined as the minimum magnetic flux needed to elicit a threshold EMG response (≥ 50 mV in peak-to-peak amplitude) in a resting target muscle (abductor pollicis brevis) in 5/10 trials using single-pulse TMS administered to the contralateral hand area of primary motor cortex.

TMS coil positioning for treatment will be individualized to account for individual differences in physiology, which are prominent in developmental samples. We will create individual finite element method (FEM) models for each participant using SimNIBS (45) and the participant’s baseline anatomical MRIs (46–48). Models will identify the coil location and orientation (x, y, z coordinates) that shows the highest correlation between the modeled electric field magnitude and the positive z-values of fMRI activation from the finger tapping task, as in Baynel et al. (49).

TMS parameters are as follows: 1) 1 Hz protocol: single train of 2000 pulses at 110% RMT (33 min duration), 2) cTBS protocol: bursts of 3 pulses at 30 Hz repeated every 200ms (5 Hz burst frequency), single uninterrupted 40 sec train, 600 total pulses at 90% resting motor threshold (40 sec duration), 3) sham protocol: to enhance masking, half of sham participants will be exposed to the 1 Hz sequence and half will be exposed to the cTBS sequence.

#### CBIT protocol.

We will follow the published CBIT manual (6). Participants will receive 10 sessions of CBIT, delivered daily on weekdays for two weeks. The manual specifies content for 8 sessions but is intended to be flexibly delivered, such that competing response-focused sessions can be increased in number. The manual has previously been delivered in intensive (daily) formats (50, 51). CBIT consists of the following components: 1) psychoeducation about tics; 2) functional interventions (behavioral strategies to decrease the impact of tic-exacerbating factors); 3) competing response training; and 4) social support to bolster skills use. Given the premise for pairing CBIT and TMS, the CBIT protocol for the current study will emphasize daily competing response training and therapist-supported practice. Competing responses will be taught in a graded manner, beginning with most distressing tics. Session 1 will include psychoeducation and creation of the tic hierarchy and sessions 2–10 will focus on competing response training. Competing response practice will be assigned for between-session homework, and completion will be tracked via daily session forms.

#### Criteria for discontinuing or modifying allocated interventions {11b}

rTMS/cTBS stimulation intensity may be adjusted to improve tolerability. All such adjustments will be reviewed by a study physician and documented.

Anticipated circumstances under which participants will be withdrawn from the research without their consent include: 1) the participant no longer meets inclusion/exclusion criteria; 2) study investigators decide that the participant has an emotional, physical, or behavioral reaction that poses a safety concern or interferes with data collection (e.g., poor compliance with instructions, too much anxiety to comfortably proceed with a task); 3) significantly deteriorating clinical course (e.g., emergence of acute suicidality); 4) significant adverse reaction to TMS; or 5) serious physical illness. Consistent with informed consent procedures, participants will be free to decide to withdraw at any time for any reason.

### Strategies to improve adherence to interventions {11c}

During TMS delivery, coil placement and orientation will be continually monitored using a stereotaxic neuronavigation system (BrainSight 2.3.5, Rogue Research, Montreal, Quebec, Canada). To ensure CBIT protocol adherence, sessions will be video recorded and a randomly selected 20% will be rated by a psychologist with expertise in CBIT using established compliance forms. Patient CBIT compliance will be tracked by the therapist at each session.

### Relevant concomitant care permitted or prohibited during the trial {11d}

To increase external validity of findings, we will include participants taking psychotropic medications that have been stable for 6 weeks and expect to remain stable for the approximately 3-week treatment protocol. Those who previously received tic-specific therapy will be included if they meet the tic severity criterion. Youth receiving other forms of psychotherapy will be included provided these treatments are not focused on tics. All concurrent treatments will be monitored during the study period.

### Provisions for post-trial care {30}

The study does not include specific provisions for ancillary or post-trial care. Referrals for clinical care will be provided to participants/parents who ask for this information. If licensed clinical study staff feel that a participant is likely to benefit from additional clinical care for tics or another diagnosis, participants/parents will be given relevant recommendations and referrals.

### Outcomes {12}

Study assessment measures are listed in Table 1.

## Phase 1

Primary outcomes focus on neural change. The primary measure will be within-subject change in activation within SMA from pre- to post-treatment, as assessed by participant blood oxygenation level dependent (BOLD) signal during the fMRI motor task (bilateral finger tapping). The secondary measure of neural target engagement will be within-subject change in RSFC of SMA-mediated brain circuits from pre-to post-treatment (SMA-DLS, SMA-M1).

We will also examine several other outcomes. First, we will evaluate within subject change in tic controllability from pre- to post-treatment as measured by the TST. Second, we will assess safety and feasibility, as measured at baseline, post-treatment, daily treatment sessions, and 1- and 3-month follow ups using staff-administered forms, aggregated as the number of treatment-related adverse events and tolerability ratings of side effects. Finally, we will explore between group differences on measures of clinical functioning from pre- to post-treatment and over the 1- and 3-month follow-up period (Yale Global Tic Severity Scale, Premonitory Urge for Tics Scale, Child/Adult Behavior Checklist, Sheehan Disability Scale, Behavior Rating Inventory of Executive Functioning) and treatment satisfaction (Client Satisfaction Questionnaire).

## Phase 2

The primary outcome is within-subject change in RSFC task activation of SMA-mediated brain circuits from pre-to post-treatment and its relationship to 1) treatment group assignment, 2) within-subject change in TST-measured tic controllability, and 3) within-subject change in the Total Score of the YGTSS. Secondary analyses will 1) describe the trajectory of change in tic controllability across daily treatment sessions and 2) explore the durability of change in RSFC of SMA-mediated circuits through the 1-month follow-up and clinical outcomes through 6-month follow-up.

### Participant timeline {13}

Participant-facing activities are depicted in Fig. 1. Participants who meet initial eligibility screening criteria (on phone screening, and in some cases, also the optional detailed eligibility assessment) will be scheduled for the “pre-treatment assessment,” at which time consent/assent and pre-treatment measures and MRIs will be completed. Daily sessions of CBIT + TMS will begin within 10 calendar days of the pre-treatment assessment, with a targeted window of 2–3 business days (to allow for runtime and checking of targeting pipeline). Participants will complete 10 CBIT + TMS visits within 13 business days. The post-treatment visit will be completed within the 10 calendar days following the last CBIT + TMS visit, with a targeted window of 1–3 business days. Post-treatment follow-up MRI scans will occur at least 24 hours after the last TMS session to ensure that observed neural activity is not simply the acute after effect of TMS. The 1-month assessment will be completed 4–6 weeks after the last CBIT + TMS visit, and the 3-month assessment will be completed 12–14 weeks after the last CBIT + TMS visit. In Phase 2, the additional 6-month assessment will be completed 24–26 weeks after the last CBIT + TMS visit.

### Sample size {14}

Power analyses were calculated for our sample sizes (Phase 1 = 60, Phase 2 = 60) assuming 20% attrition (Phase 1 = 48, Phase 2 = 48). A sample of N = 48 gives us 80% power to detect an effect size of at least η^2^ = 0.18 for differences in SMA activation between active TMS and sham and least η^2^ = 0.18 for differences in RSFC between active TMS and sham. This sample will give us 80% power to detect an effect size of at least d = 0.90 for a within-subject improvement in tic controllability in Phase 1 and an effect size of at least d = 0.58 to test change in tic control trajectory across groups in Phase 2. For Phase 2’s exploration of durability of change, using simulations, assuming a moderate correlation of 0.5 on the RSFC or the clinical outcomes within a participant across the times of assessment, we have at least 80% power to detect a standardized difference of at least d = 0.71 between the treatment and sham groups on functional connectivity or the functional outcomes.

### Recruitment {15}

Recruitment strategies will include dissemination of study information to clinicians within the university-affiliated MHealth Fairview hospital system and in community practices across the region, focusing on those clinicians most likely to encounter individuals with tics (i.e., neurologists, psychiatrists, primary care practitioners). We will use an IRB-approved process to send messages to potentially eligible patients via the MHealth Fairview electronic medical record system. Flyers describing the study will be distributed in public physical spaces (e.g., libraries, community events) and posted on departmental websites and lab social media accounts.

## Assignment of interventions: allocation

### Sequence generation {16a}

Block randomization, stratified on baseline TST performance (tic controllability below or above 50%) and medication status (on vs. off), with equal block sizes will be carried out using the blockrand package in R(52). For Phase 1, the blocks are of size 6, designed in a manner so that the odds of allocation to 1Hz rTMS vs. cTBS is 1:1, and the odds of allocation to active vs sham is 2:1. For Phase 2, the blocks are of size 2, designed so odds of allocation to active vs. sham are 1:1. The randomization key is a digital file stored in a place only accessible to unmasked staff (TMS supervisor and statistician).

### Concealment mechanism {16b}

Conditions are concealed within the digital randomization file.

### Implementation {16c}

Study staff will send stratification information obtained from the pre-assessment visit to the study statistician via a REDCap form. After the statistician has completed the randomization, the TMS supervisor verifies the randomization. TMS operators are informed of protocol type (1 Hz or cTBS) and coil (where active vs. sham coils are concealed with coded letters, i.e., A or B). A separate active coil is used for RMT determination to enhance coil masking.

## Assignment of interventions: Blinding

### Who will be blinded {17a}

Procedures will be implemented to control for expectancy effects related to TMS stimulation. Persons who will be masked to TMS status (active vs. sham) are: participants, parents (if applicable), and study staff administering clinical assessments, coding TST videos, delivering CBIT therapy, and collecting the MRI data. Unmasked personnel will be the study statistician and the TMS operator supervisor. The TMS operator, participant, and parents will be masked to active vs. sham status in Phase 1 but will be aware of 1Hz vs. cTBS allocation; they will be fully masked in Phase 2. Staff who administer clinical rating scales and code TST videos will not be present for a given participant’s CBIT + TMS visits to ensure masking to overall therapy progress. At post-treatment, forms assessing masking adequacy will be given to participants, parents, and masked staff who conduct CBIT sessions and assessments visits.

### Procedure for unblinding if needed {17b}

Unmasking of TMS status will occur in situations where staff deem this necessary for participant safety, to address a technical issue related to the TMS device, or another unforeseen situation in which TMS status is critical for study conduct. Unmasking will be limited to those individuals deemed most critical for addressing the inciting situation. Independent Evaluators (i.e., staff responsible for conducting pre-, post-, and follow-up assessments with participants) will not be intentionally unmasked to ensure that clinical functioning can be tracked by a staff member who is otherwise unfamiliar with the course of treatment.

### Data collection and management

#### Plans for assessment and collection of outcomes {18a}

Clinical assessment measures and timing are listed in Table 1. The clinician conducting the pre-, post-treatment, and follow-up assessments will be an Independent Evaluator (IE) masked to all treatment-related information. IEs will be l research staff at least at the BA/BS-level trained to criterion on all study measures who meet weekly with a licensed clinician supervisor for criterion maintenance. Assessments will be video-recorded and a random 20% will be reviewed to prevent drift.

Treatment measures will include separate daily CBIT and TMS session notes, the TMS Adverse Events Questionnaire, and the Concomitant Medications and Therapy Tracking Form, which will be completed by the relevant staff member (TMS operator or CBIT therapist). All clinical assessment and treatment measures will be entered into REDCap and double checked for completion and accuracy by a second staff member within 2 business days of the study visit.

##### Tic Suppression Task (TST).

In this paradigm, a participant is seated alone in a room (to reduce observation reactivity effects (53)) in front of a GoPro video camera and computer that provides condition instructions. The TST will consist of 2, 3 min conditions: 1) rest: youth is instructed to stay seated and tic freely, a measure of naturally occurring tic frequency; 2) suppression: youth is instructed to suppress tics. Participants are prompted every 30 sec to verbally state a premonitory urge using a visual 0–10 scale. Coding. Following established tic coding steps (54), videos will be coded using DataVyu(55) to establish tic frequencies (tics per minute for each condition). Coders will be masked to time (i.e., whether clip is pre- or post-treatment), TMS status, and TST condition. An independent rater will code 20% of videos to establish interrater reliability. Reliability will be calculated as percent agreement for each video clip, where agreement will be defined as a code of tic occurrence within a 1 sec tolerability window. The primary rater’s coding will be considered valid with ≥ 80% agreement; clips with lower agreement will warrant additional training and re-coding. Final interrater reliability for all videos will be calculated using intraclass correlation coefficients and Cohen’s kappa (56). The primary TST outcome will be tic controllability, calculated as a percent change score [(“Free to Tic” tic frequency - Suppression tic frequency)/ “Free to Tic” tic frequency*100] (14). Positive values indicate tic reduction during suppression (i.e., better suppression), near-zero values indicate little to no difference between conditions (i.e., poor suppression), and negative values indicate tic increase during suppresision (i.e., tic worsening during suppression condition). We will calculate this value for each administration of the TST.

##### MRI and fMRI data acquisition.

A Siemens Prisma 3T scanner will be used for image acquisition. To minimize head motion during the scan, we will immobilize the participant’s head with foam wedges that fit snugly between the head and 32 channel receive only head coil. We will implement real-time monitoring of subject motion during all fMRI scans using Framewise Integrated Real-Time MRI Monitoring (FIRMM, (57, 58) allowing us to scan to criterion (at least 5 min of usable data). As needed, we will re-run scans or bring back participants for a repeat scan session. We will acquire scans based on the ABCD acquisition protocol and pulse sequences:

1. Structural scans for anatomical reference, including a) whole brain 3D sagittal T1 weighted inversion prepared RF-spoiled gradient echo scan which includes motion-driven selective reacquisition of k-space to compensate for subject motion (TR = 2500ms, TE = 2.9ms, TI = 1070ms, 1.0mm isotropic voxel, flip angle = 8 degrees (7 min)) and b) whole brain 3D sagittal T2 weighted variable flip angle fast spin echo scan which includes motion-driven selective reacquisition of k-space to compensate for subject motion (TR = 3200ms, TE = 565ms, 1.0mm isotropic voxel, variable flip angle (6 min)).
2. Resting fMRI: Whole brain acquisition using the ABCD SMS sequence (60 axial slices, 2.4mm isotropic voxel size, TR = 800ms, TE = 30ms, FOV = 216mm, matrix = 90×90, MB factor = 6, 383 volumes (5 min)). During this scan participants will be instructed to keep their eyes open and orient to a fixation cross. Four runs of the resting scan will be collected during each visit.
3. Spin Echo fMRI reverse phase encode scan pair: Whole brain acquisition using the ABCD SMS spin echo sequence (60 axial slices, 2.4mm isotropic voxel size, TR = 7030ms, TE = 80ms, FOV = 216mm, matrix = 90×90, 1 volume pair (0.5 min))
4. Finger Tapping Task, for functional localization of SMA. Whole brain acquisition using ABCD SMS sequence (60 axial slices, 2.4mm isotropic voxel size, TR = 800ms, TE = 30ms, FOV = 216mm, matrix = 90×90, MB factor = 6, 394 volume (5.5 min)). The task involves alternating 15sec blocks of simultaneous bilateral finger tapping and rest, cued by visual stimuli. This task is used to isolate neural activity in motor planning and execution areas, including SMA, and is a highly reliable, well-established fMRI task used for motor mapping (59) and for TMS targeting of motor cortex and SMA by our group and others.(32)

##### MRI and fMRI Pre-Processing.

The ABCD-BIDS preprocessing pipelines will be applied to the structural T1 and T2 data as well as the resting state data (60). We will use established, rigorous approaches for mitigating head motion artifact (61–63) that have been shown to be effective for studies of group or individual differences (64, 65).

##### Tasks for TMS coil placement.

Processing and analysis for finger tapping task data will also be carried out using the ABCD-BIDS processing pipeline. We will conduct a whole-brain linear regression analysis with the contrast of interest rest vs. active tapping, yielding a whole-brain activation map for each person. Activation within the SMA will be mapped onto the subject’s T1, the weighted centroid for the activation will be computed and this coordinate will be used for coil placement in the individual electric field models

#### Plans to promote participant retention and complete follow-up {18b}

Efforts will be made to reduce barriers to attending study visits, including offering study visits at preferred times, sibling care during study visits, and remote options for completion of study tasks that do not need to be conducted in-person (e.g., clinical assessments). We will use an Adjunctive Services and Attrition Plan (ASAP) to address any situations that require intervention by study staff beyond that afforded by the assigned treatment condition. Participants will be allowed up to 1 ASAP session during the acute treatment phase and 1 during the follow-up period. Participants will be compensated for all study activities, with increasing amounts of compensation in the follow-up period to promote retention.

For participants who discontinue or deviate from intervention protocols, we will aim to complete at least one final clinical assessment. We will attempt to provide participants with appropriate care referrals in the event of any withdrawals or drop-outs.

### Data management {19}

This study will utilize the secure, web-based Research Electronic Data Capture (REDCap) system for data input. Range checks for data values and missing data will be automatically flagged in REDCap. All REDCap data will be double checked by a second staff member to ensure data integrity. Imaging data will be deidentified and stored on secure servers. TST and visit videos will be stored on Box, a HIPAA-compliant file storage platform, and analyzed on secure computers/servers. Access to password-protected databases will be limited to the investigators and trained staff listed on the study IRB, in accordance with institutional policy. Data file archiving and back-up will be performed on a regular basis.

### Confidentiality {27}

All members of the project will maintain up-to-date certification on research participant confidentiality and privacy through the Collaborative Institutional Training Initiative (CITI) curriculum. Study staff will additionally participate in HIPAA and PHI training through UMN. All data will be identified and labeled only by subject ID numbers, which will be stored separately from the identifying information and from consent and assent forms.

#### Plans for collection, laboratory evaluation and storage of biological specimens for genetic or molecular analysis in this trial/future use {33}

Not applicable, as no biological specimens are collected.

## Statistical methods

### Statistical methods for primary and secondary outcomes {20a}

#### Phase 1

##### Primary outcome (SMA activation):

An ROI analysis will be performed to determine the time*treatment group differences in SMA activation elicited by the fMRI motor task (finger tapping). The SMA ROI will be identified by placing a seed (sphere with 5mm radius) that matches the coordinate used for individual TMS coil placement. Activation parameters in the SMA will be identified in a first-level analysis, using a general linear model (GLM) to model each subject’s SMA time course during the motor task. These activation parameters will be used in a subsequent linear mixed effects (LME) model. The predictors of the LME will include the baseline activation, group indicators for the stimulation groups, and baseline x group interactions. Random effects will be used to model within-subject variation from the first-level analyses. Altogether, the LME will allow us to model how the within-subject change in activation from pre-treatment to post-treatment differs across active and sham conditions.

##### Secondary outcome (SMA connectivity):

We will use a seed-based analysis approach to test RSFC between a priori ROIs and use a false discovery rate (FDR) correction for multiple comparisons threshold of *p* < 0.05. Functional connectivity analyses will be conducted by placing a seed (sphere with 5mm radius) in each ROI. SMA seeds will match coordinates used for individual TMS coil placement. We will similarly individually identify ROI seeds for left and right primary motor cortex using finger tapping task data. Right and left DLS (i.e., putamen) ROIs will be individually anatomically based and identified using FreeSurfer anatomical masks on each participant’s T1 volume aligned to each participant’s rs-fMRI data using bbregister (66), mirroring our prior work (67). Average BOLD time-series data will be extracted from each ROI, for each participant, and used to calculate correlation coefficients with SMA. These will be converted to individual Z-scores using Fisher’s transformation, yielding the indices representing RSFC between the seed and each of the targets (4 connections). ANOVA will be used to model how the within-subject change in functional connectivity from pre-treatment to post-treatment differs across active and sham conditions. Effect sizes from the ANOVA will be computed to determine target engagement.

In addition, we will also investigate the effects of each TMS regimen on the connectivity in other nodes of CSTC circuits. To this end, we will extract the ROI-level time courses from each region in the CSTC, and we will use partial correlations to obtain an estimate of connectivity between each ROI pair within the CSTC while accounting for the data observed in the other ROIs. Given the collection of partial correlations for each study participant, we will use the sum-of-powers (SPU) test, which uses the collection of partial correlations simultaneously, to assess for group differences across the sham and TMS regimens (68). While changes in SMA-M1 or SMA-DLS will suffice to satisfy Milestone 1 in our *Go Criteria*, testing additional functional connections will broaden our investigations into the CSTC when assessing the target engagement of each regimen.

##### Other outcomes.

TST “tic controllability” scores (14) will serve as the primary outcome in an ANOVA model, which will be used to establish if there exist differences in how the 1Hz, cTBS, and sham conditions affect change in tic suppression ability from pre- to post-treatment. Group indicators for these conditions will be the primary predictors for the model.

#### Phase 2l

##### Primary outcome (SMA connectivity and its relationship to clinical outcomes):

Analysis will be conducted using ANCOVA. Primary predictors are group indicators for sham and active TMS. Dependent variables are the differences in global tic severity (YGTSS) pre- and post-treatment. Differences pre- and post-treatment in RSFC and its interaction with the group indicators will be included as potential mediators for the dependent variable.

As in Phase 1, we will use a seed-based analysis approach to measure RSFC between a priori ROIs (SMA-DLS and SMA-M1). However, it is possible that change will occur outside of these a priori selected networks. Thus, we will also conduct a more comprehensive network analysis. For each subject we will measure functional connectivity by calculating interregional partial correlations across a set of 300 ROIs that comprehensively samples cortex, subcortex, and cerebellum (69). This set of 300 ROIs can be broken down into a number of functional brain networks (e.g., fronto-parietal, cingulo-opercular, default-mode, somatomotor). Given the large number of ROIs relative to the proposed sample size, to estimate the partial correlations we will use the graphical lasso (70), which is a reliable approach for estimating partial correlations (71). Finally, we will average the correlations within nodes of a network and between nodes of different networks to yield composite scores reflecting intra-network and inter-network integrity (72). ANOVA will be used to model how the within-subject change in functional connectivity from pre-treatment to post-treatment differs across active and sham conditions. Effect sizes from the ANOVA will be computed to determine target engagement.

##### Secondary outcome (trajectory of change in tic controllability):

We will identify the potentially non-linear relationship between the number of treatment days for each subject and their change in tic controllability on the TST. We will fit different dose-response models using non-linear least squares, and then identify the best fitting model using Akaike (AIC) or Bayesian information criteria (BIC; (73)). Using the fitted dose-response curve, we will find the lowest number of days that leads to 90% of the maximum effect (74).

##### Secondary outcome (intervention durability):

Durability of changes in SMA-M1 and SMA-DLS functional connectivity and clinical outcomes will be measured through 1 month follow-up. Linear mixed models (LMMs) will be used to model the trajectories of each of the functional connectivity measures and clinical outcomes across the times of assessment. Durability will be assessed by quantifying the role of time in each of the functional connectivity and clinical outcomes.

#### Interim analyses {21b}

No interim analyses are pre-specified for this trial. As part of performing risk/benefit assessments, the study Data Safety Monitoring Board (DSMB) can request interim efficacy information in addition to available safety data. The study does not have any formal stopping rules. The DSMB votes on study continuation at each meeting.

### Methods for additional analyses (e.g. subgroup analyses) {20b}

None planned.

#### Methods in analysis to handle protocol non-adherence and any statistical methods to handle missing data {20c}

We will carry out the analyses of the data according to the intention-to-treat principle. We will compare baseline characteristics of those who drop out vs. those who do not and adjust models based on significant predictors of dropout. For those who drop out during the follow-up period or miss some assessments during follow-up, we will use multiple imputation by chained equations (MICE) to allow for missing data without loss of those cases from the model under the assumption of Missing at Random (MAR). If dropout status appears to be Missing Not At Random (MNAR), i.e., dropout is associated with an unobserved outcome, we will run sensitivity analyses using MICE to compare imputed and non-imputed models results to assess robustness of statistical inference.

#### Plans to give access to the full protocol, participant level-data and statistical code {31c}

Participant-level de-identified data will be submitted to the National Institute of Mental Health Data Archive (NDA, Collection #3650). Participants can opt into joining a videographic data registry that is held by the PI; these identifiable videographic can be shared with qualified researchers at NIH-recognized research institutions via a rigorous approval system, in accordance with our IRB and institutional guidelines. R code for statistical analyses can be made available upon reasonable request. The MRI pdf and/or exar1 scanning protocol can be made available upon request.

## Oversight and monitoring

### Composition of the coordinating centre and trial steering committee {5d}

Study governance for this single-site study is organized into “teams” with specific responsibilities, including the Oversight Team, Recruitment Team, Intervention Deployment Team, Data Management Team, Neuromodulation Team, and Neuroimaging Team. The Oversight Team is led by the PI and is responsible for global oversight of study conduct, procedural and scientific integrity, regulatory management, data quality assurance, data analysis, and dissemination of findings. Each team is led by a doctoral-level Co-Investigator. Each team works with the Oversight Team to develop and monitor standard operating procedures. Each team has a weekly meeting with the PI focused on decisions and progress within their scope of responsibility. Full study meetings are held quarterly and as-needed.

### Composition of the data monitoring committee, its role and reporting structure {21a}

An independent DSMB has been convened for this study. Members must be independent of any conflict of interest with the research project and study investigators, in accordance with NIMH policy (75). Members include a biostatistician with expertise in randomized clinical trial methodology, a researcher with expertise in neurodevelopmental disorders, and a child/adolescent psychiatrist. A study-specific DSMB charter was created prior to enrollment of the first participant. The DSMB is advisory to the PI. The PI holds ultimate responsibility for decisions regarding the trial.

The DSMB will meet annually to 1) monitor study safety, quality, and conduct and 2) decide whether adequate participant safeguards are in place. The DSMB will review: 1) study progress, including assessments of data quality and participant recruitment, accrual, and retention; 2) outcome and adverse event data, to determine whether there is any change to the anticipated benefit-to-risk ratio; 3) relevant external information that may have an impact on study ethics or participant safety; and 4) study procedures for privacy and confidentiality. The DSMB will review de-identified reports annually. At each meeting, the DSMB will vote to continue the trial unchanged, continue the trial with modifications, or terminate the trial. The DSMB may also make recommendations about other aspects of the trial such as the recruitment of participants and the conduct of the trial.

At any time during the trial, regulatory authorities and any other body or individual involved with the conduct of the trial may seek the advice of the DSMB about any concern that they may have about the conduct, outcome, or continuation of the trial. Any such requests are directed to the DSMB Chairperson.

### Adverse event reporting and harms {22}

Study staff will formally assess for adverse events at each post-randomization study visit using the comprehensive study adverse events questionnaire. All adverse events are reviewed by the PI. Per the DSMB charter, key events include: 1) seizures, 2) side effects to the study treatment, 2) hospitalization, 3) suicidality, and 4) premature drop-out from treatment. Data will indicate *likely, possible* or *unlikely* relation to study interventions.

Any unexpected serious adverse events or unanticipated problems involving risks to participants that are possibly related to rTMS will be promptly reported to the Chair of the DSMB, IRB, FDA, and assigned NIMH Project Officer in accordance with relevant rules and regulations. Investigators will submit yearly progress reports to IRB and FDA summarizing data and safety monitoring activities, including adverse events deemed expected or unrelated to the study and protocol deviations that do not affect the scientific soundness of the research plans or the rights, safety or welfare of participants.

### Frequency and plans for auditing trial conduct {23}

The NIMH CTOB Clinical Research Education, Support, and Training Program (CREST) will provide ongoing regulatory monitoring. Planned monitoring visits will occur upon study initiation, yearly, and after completion of data collection. The monitoring schedule may be revised based on considerations such as accrual rate, protocol deviations, magnitude of data corrections required, or DSMB recommendation.

### Plans for communicating important protocol amendments to relevant parties (e.g. trial participants, ethical committees) {25}

Protocol changes will not be implemented without prior IRB approval. Investigators will follow FDA regulations that govern significant risk device investigations (76). These require FDA review and approval prior to making significant changes in the study protocol or in informed consent that may increase participant risk or impact the scientific soundness of the study. Protocol changes will be indicated on ClinicalTrials.gov. If any amendments result in changes to informed consent, participants who have not completed the study will be re-consented/assented.

### Dissemination plans {31a}

Results will be submitted to ClinicalTrials.gov via the Protocol Registration and Results System Information Website. Informed consent will have a specific statement informing participants that results from the study will be posted on ClincalTrials.gov.

## Discussion

The present CBIT + TMS trial is designed to test whether augmenting CBIT with inhibitory, noninvasive stimulation of SMA via TMS procedures modifies activity in SMA-mediated circuits and enhances tic controllability in young people with tic disorders.

This trial is the first test of combined brain stimulation and CBIT focused on comparing brain stimulation strategies. The results will provide insight into whether brain stimulation can augment CBIT, a goal that is important, given that 50% of patients do not benefit from CBIT alone. Collection and analysis of multimodal data (neural, behavioral, clinical) will inform our understanding of the mechanisms underlying tic etiology, maintenance, and treatment response. The study applies a functional targeting approach, wherein the two intervention modalities are matched based on the premise that TMS and CBIT-elicited behaviors are synergistically engaging common neurocircuitry (i.e., CBIT and the TMS protocols are both engaging sensorimotor circuitry to support tic control behavior). As we have articulated elsewhere (77), TMS approaches to augment cognitive-behavioral therapies are most likely to be positively synergistic when the interventions activate common, complementary, or compensatory circuits that support CBT-elicited behaviors.

Notably, this study will be one of few clinical trials to date examining TMS as an intervention for a pediatric sample. TMS is a potentially promising modality for targeting aberrant brain circuitry involved in developmental neurological and psychiatric illnesses. TMS in children has been demonstrated to be safe, comfortable, well-tolerated, and it has minimal side effects (78). This study will add to our understanding of pediatric TMS efficacy, tolerability, and safety.

### Limitations and Anticipated Challenges

Tic-related excessive and uncontrollable movements can present challenges to fMRI data collection and TMS administration. We are implementing several processes to mitigate this challenge. First, for MRI data acquisition, we will employ a tool (FIRMM) for assessing real-time movement. This will enable us to determine if data meet our motion quality criteria in real time, so we can adjust, extend, or repeat scanning visits (57, 58). Additionally, we will use established strategies for obtaining high-quality fMRI data from youth with tics(58) and data-processing procedures to adjust for motion (62, 63). For TMS administration, we have worked with Child Life specialists in our institute and used patient and parent feedback to implement measures that increase comfort and tolerability (e.g., use of supportive pillows and/or a weighted blanket to stabilize the body, allowing participants to bring comfort object). TMS coil placement will be continually tracked with the Brainsight software, and staff will document the percentage of “on target” pulses and participant-rated efforts to actively suppress tics during stimulation. Our study will enable us to explore the effectiveness of these strategies and methodologies for use in future study designs.

Although previous CBIT efficacy trials (8, 9) implemented CBIT in 8 sessions over 10 weeks, we will conduct treatment sessions daily versus weekly to maintain consistency with TMS protocols and key principles (79–81). Although this results in CBIT administration that deviates from typical outpatient setting protocols, CBIT was designed to be delivered flexibly, and studies that have examined flexible delivery have found it to be feasible and effective (50, 51). Comparing our observed effect size for tic severity (YGTSS) change to those reported in other CBIT efficacy studies will help us understand the extent to which this format is comparable. Phase 2 analyses examining the trajectory of change in tic controllability across treatment will also inform our understanding of dose-response relationships. Finally, session-level data collection will be examined retrospectively to explore whether certain CBIT process elements may relate to outcomes in this format (e.g., number of tics targeted in treatment, homework compliance).

A final limitation worth noting is the intensive scheduling requirements, such that participant burden for the study is elevated for the intervention portions of each phase. Unanticipated schedule changes may affect protocol timing. To mitigate these challenges, we will provide a high level of support from research staff surrounding scheduling logistics, plan for maximum scheduling flexibility (e.g., after business hours and offering TMS visits on weekends as necessary) and provide schedules that are planned around breaks (e.g., school break in the summer). We will document feedback from those who choose to participate as well as those who decline participation to understand the extent to which the intervention schedule may impact accessibility.

## Trial status

This report is based on Protocol Version 6 (February 24, 2022). Recruitment began December 2020. Recruitment pace was slower than anticipated due to circumstances and restrictions related to the COVID-19 pandemic. Recruitment for Phase 1 is expected to be complete by January 2024. If Phase 1 milestones are met, Phase 2 is anticipated to begin shortly after Phase 1 completion and to have a duration of 3 years.

## Figures and Tables

**Table 1:** Study assessment measures in both phases.

Assessment	Purpose	Reporter	Administration Time				
Measure			Screen	Pre- Tx	Sessions	Post- Tx	Follow- Up
Screening (52,53)	Eligibility and safety screening	AP or P	X				
Medical records	Current medications, relevant/available documents; for eligibility determination	AP or P	X				
Demographics	Sample characteristics	AP or P		X			
Wechsler Abbreviated Scale of lntelligence(54)	Eligibility	AP or CP		X			
MINI 7 KID (55) or adult (56)	Structured diagnostic interview	IE		X			
Yale Global Tic Severity Scale, YGTSS(57)	Measure of tic severity and checklist of specific tic types	IE		X		X	X
Tic Suppression Task, TST (14,58)	Direct observation measure of tic controllability	AP or CP		X	X	X	X
Parent/Adult Tic Questionnaire(59,60)	Measure of tic symptoms and severity	AP or P		X		X	X
Premonitory Urge for Tics Scale (61,62)	Measure of intensity of urges to tic	AP or CP		X		X	X
Child/Adult Behavior Checklist (63,64)	Measure of broad emotional and behavioral functioning	AP or P		X		X	X
Sheehan Disability Scale (65)	Measure of functional impairment	AP or CP&P		X		X	X
Tic Impact questions	Measures of how tics impact daily function	AP or CP&P		X		X	X
Behavior Rating Inventory of Executive Function (66)	Measure of impairment of executive function	AP or CP&P		X		X	X
Ask Suicide- Screening Questions (ASQ)	Suicidality screen	IE		X		X	X
TMS Adverse Effects Questionnaire (53)	Tracks side effects of TMS	Staff IE			X	X	X
Clinical Global Impressions (CGI)	Global measure of illness severity and improvement	IE AP or CP P		X		X	X
Stimulation context tracking form	Participant behavior and emotional state during rTMS stimulation	Staff			X		
Masking form	Assess belief about whether participant got active or placebo TMS	AP or CR R IE, Staff				X	
Concurrent Treatment Tracking Form	Record of all medications and dosages, psychotherapy	Staff		X	X	X	X
Client Satisfaction Questionnaire (67)	Assess patient satisfaction with the intervention	AP or P				X	X

Abbreviations: AP = adult participant, CP = child participant, IE = independent evaluator, P = parent, Tx = treatment

## Availability of data and materials {29}

Data will be submitted to the NDA every 6 months and held privately until shared with the research community. In accordance with NIMH expectations at the time of the funding award, “data will be shared with the research community when papers using the data have been accepted for publication or at the end of the award period, whichever is sooner” (82). Specific data used for each resulting publication will be shared as NDA studies when papers are accepted for publication.

## Abbreviations

CBIT: Comprehensive Behavioral Intervention for Tics
CSTC: Cortico-Striatal-Thalamo-Cortical Circuit
SMA: Supplementary Motor Area
M1: Primary Motor Cortex
RSFC: Resting State Functional Connectivity
DBS: Deep Brain Stimulation
rTMS/TMS: Repetitive/Transcranial Magnetic Stimulation
cTBS/TBS: Continuous/Theta Burst Stimulation
fMRI/MRI: Functional/Magnetic Resonance Imaging
DSMB: Data Safety Monitoring Board
IRB: Institutional Review Board
FDA: United States Food and Drug Administration
NDA: National Institute of Mental Health Database
PI: Principal Investigator
RMT: Resting Motor Threshold
FEM: Finite Element Method
TST: Tic Suppression Task
FIRMM: Framewise Integrated Real-Time MRI Monitoring
IE: Independent Evaluator
ASAP: Adjunctive Services and Attrition Prevention
REDCap: Research Electronic Data Capture
CITI: Collaborative Institutional Training Initiative
ROI: Region of Interest
GLM: General Linear Model
LME: Linear Mixed Effects
FDR: False Discovery Rate
MAR: Missing At Random
MNAR: Missing Not At Random
LMM: Linear Mixed Models
MICE: Multiple Imputation By Chained Equations
NIMH: National Institute of Mental Health
CREST: Clinical Research Education, Support, and Training Program
CNBD: Center for Neurobehavioral Development
NNL: Noninvasive Neuromodulation Laboratory
CMRR: Center for Magnetic Resonance Research
